# A Microfluidic In Vitro Three-Dimensional Dynamic Model of the Blood–Brain Barrier to Study the Transmigration of Immune Cells

**DOI:** 10.3390/brainsci12101293

**Published:** 2022-09-25

**Authors:** Megha Meena, Robin Vandormael, Maxime De Laere, Isabel Pintelon, Zwi Berneman, Regan Watts, Nathalie Cools

**Affiliations:** 1 Laboratory of Experimental Hematology, Vaccine & Infectious Disease Institute (VAXINFECTIO), Faculty of Medicine and Health Sciences, University of Antwerp, 2610 Antwerp, Belgium; 2 Department of Product Development, Faculty of Design Sciences, University of Antwerp, 2000 Antwerp, Belgium; 3 Center for Cell Therapy and Regenerative Medicine, Antwerp University Hospital, 2650 Edegem, Belgium; 4 Laboratory of Cell Biology and Histology, Faculty of Pharmaceutical, Biomedical and Veterinary Sciences, University of Antwerp, 2610 Antwerp, Belgium

**Keywords:** blood–brain barrier, central nervous system, endothelial cells, TripleB slides, microfluidic device, transendothelial electrical resistance, FITC-dextran permeability, P-glycoprotein

## Abstract

To study the biodistribution of new chemical and biological entities, an in vitro model of the blood–brain barrier (BBB) may become an essential tool during early phases of drug discovery. Here, we present a proof-of-concept of an in-house designed three-dimensional BBB biochip designed by us. This three-dimensional dynamic BBB model consists of endothelial cells and astrocytes, co-cultured on opposing sides of a polymer-coated membrane under flow mimicking blood flow. Our results demonstrate a highly effective BBB as evidenced by (i) a 30-fold increase in transendothelial electrical resistance (TEER), (ii) a significantly higher expression of tight junction proteins, and (iii) the low FITC–dextran permeability of our technical solution as compared to a static in vitro BBB model. Importantly, our three-dimensional BBB model effectively expresses P-glycoprotein (Pg-p), a hallmark characteristic for brain-derived endothelial cells. In conclusion, we provide here a complete holistic approach and insight to the whole BBB system, potentially delivering translational significance in the clinical and pharmaceutical arenas.

## 1. Introduction

Neurological disorders, such as multiple sclerosis (MS), Alzheimer’s disease and Parkinson’s disease, are increasingly recognized as some of the most prevalent disorders with high burden to the patients, their families, and society [1,2,3]. To date, neurological disorders are the third most common cause of disability and premature death in the EU and their prevalence and burden will likely increase with the progressive ageing of the European population [1]. Despite the substantial increase in the burden of neurological disorders, no cure is available yet for most of these neurological, noncommunicable diseases. To be effective, drugs must reach their target in the brain. However, the blood–brain barrier (BBB), which is mainly composed of a specialized microvascular endothelium, glial cells and pericytes, separates the central nervous system (CNS) from the rest of the body, and in doing so represents a major obstacle to the delivery of therapeutic drugs to the brain [4,5,6,7,8,9]. Indeed, while the presence of cell surface proteins, ion channels, efflux pumps, enzymes, specific receptors, and transporters on pericytes, vascular smooth muscle cells, and endothelial cells (ECs) maintain CNS homeostasis by regulating the exchange of materials between the circulation and the brain [10,11], the BBB also protects the CNS by blocking compounds from entering brain tissue, including neurotoxic components as well as pathogens. This highly specific and selective permeability of the BBB, excluding most therapeutics, results in many drugs’ inefficacy, as well as difficulty, to be translated into clinical practice. In order to study the biodistribution of new chemical and biological entities, an in vitro model of the BBB may become an essential tool to predict drug safety and/or drug efficacy during early phases of drug discovery and, hence, would allow broad-scale in vitro pharmacology profiling [12] and ultimately the development of new and effective treatments for neurological diseases.

The physical backbone of the BBB is a monolayer of endothelial cells that are connected by much tighter junctions than these in peripheral vessels [13]. Besides this, the molecular constituents of tight junctions, adherence junctions, and signaling pathways regulate the assembly of the endothelial cells [14]. However, other specific proteins are responsible for the rapid efflux of drugs from the CNS, and for the delivery of the essential nutrients and transmitters into the brain, including drug efflux transporters such as ATP-binding cassette (ABC) and P-glycoprotein (Pg-p), multidrug resistance proteins, and organic anion transporting polypeptides [7,13,15,16,17,18,19,20,21]. P-glycoprotein (Pg-p), the product of the multidrug resistance 1 (MDR1) or ABCB1 gene, is an ATP-dependent efflux transporter that tightly regulates the movement of cytotoxic molecules and drugs between the blood and the brain [22,23,24]. It is one of the most important efflux transporters, which significantly contributes to BBB function by extruding toxins and xenobiotics out of cells and limiting the influx and retention of a variety of lipophilic compounds [25,26]. Pg-p is an ATP-dependent drug transport protein which is present in high concentrations in the blood luminal membrane of the brain capillary endothelial cells that make up the BBB. This protein is known to actively transport a vast variety of hydrophobic amphipathic drugs out of the cell and plays a significant role in drug absorption and disposition [25,27,28,29]. Based on this, it was hypothesized that Pg-p is responsible for the very poor penetration of many relatively large (>400 Da) hydrophobic drugs in the brain [25,30].

To study the drug transmigration across the BBB, simplified in vitro BBB models that resemble the BBB have been developed [20,31,32]. Transwell systems are the most common and widely used cell-based in vitro models of the BBB. These are semi-permeable membranes separating a luminal and abluminal compartment [32]. Based on the evidence that interactions between endothelial cells (ECs) and astrocytes improves barrier properties [33], ECs are seeded in the transwell insert while astrocytes are grown on the underside of the transwell insert. These transwell systems of the BBB are ideal for permeability testing and binding affinity measurements and can be used to investigate transmigratory processes [34]. However, they do not uncover all the aspects of the BBB and lack the 3D in vivo organization, including direct cell–cell interactions. This results in lower values of the trans-endothelial electrical resistance (TEER), a measure of effective barrier formation, as compared to the in vivo TEER value, and in a phenotypic drift of ECs that acquire more generic endothelial cell properties [35]. This suggests that other players also contribute to the integrity of BBB [36]. For instance, these static models do not fully replicate the shear stress generated by the flow of blood, as well as other precise in vivo conditions of a human brain. In contrast, a dynamic in vitro BBB model with an incorporated flow can overcome most of the above-mentioned limitations and provide a quasi-physiological environment where ECs and astrocytes can establish a functional BBB that closely mimics the in vivo situation. Nonetheless, the currently available dynamic models are small and do not allow the collection of large particles or cells that have transmigrated across the barrier for further study. Hence, none of the currently available models are appraised as ideal, resulting in limited translational significance in the clinical and pharmaceutical arenas.

To overcome these limitations, we have devised a novel dynamic in vitro BBB model which parades several advantages over the existing in vitro BBB models, including its ease of handling and usage, and the maintenance of a precise dynamic microenvironment as observed in the human brain. Our model consists of human cerebral microvascular endothelial cells (hCMEC/D3) cocultured with primary human astrocytes under continuous laminar flow of medium, thereby mimicking the stable and meticulous in vivo BBB characteristics. We anticipate that this system could enable the predictive screening and evaluation of the ability of a drug candidate to permeate the BBB. More importantly, this device can also be used to study the permeation and migration of different cell types through a tightly formed BBB under healthy and inflammatory states. In doing so, our model can also be used to provide an understanding of the extent of BBB dysfunction during the pathogenesis of various neurological diseases.

## 2. Materials and Methods

### 2.1. Design and Fabrication

**Figure 1:**
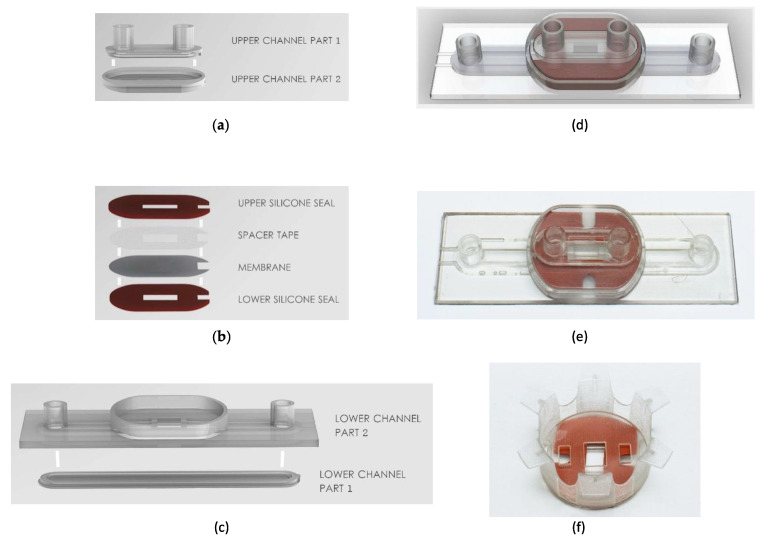
The fabrication process of the TripleB fluidic chip, beginning with the sub-assembly in (**a**) which shows the formation of the upper fluid channel (for endothial cells) comprising two rigid bodies (upper channel part 1 and upper channel 2), which are 3D printed using biocompatible MED610 resin, and are then sealed along the edges using uncured MED610 resin, which is then separately cured with a UV lamp; (**b**) then, the assembly of the removeable membrane is performed, which consists of a porous membrane with 3.0 µm pore size (Oxyphen) and spacer tape to support the delicate porous membrane structure, which are then sandwiched between an upper and lower layer of silicone rubber (Shore hardness 30 A) to ensure a tight seal between the rigid bodies of the upper and lower channels—the shapes of these components were fabricated using mechanical die-cutting and punching and exposed a surface area of 4 × 8 mm or 0.32 cm^2^ on either side of the porous membrane for the growth of the endothelial and astrocyte cells; (**c**) the formation of the lower fluid channel (for astrocyte cells) comprising two rigid bodies (lower channel part 1 and part 2). These parts are fabricated and sealed using the same method as the upper channel. These three sub-assemblies are combined together, as shown with (**d**) a render and (**e**) a photograph of the fabricated TripleB fluidic chip. A photograph of the novel transwell insert is shown in (**f**) which comprises the two rigid bodies (the flanged carrier and the cover with bayonet-type connection) and the removable membrane assembly.

We engineered novel microfluidic slides with a removable microporous membrane (3 µm pore size) that allows for the seeding of cells on the opposite side of the membrane, similar to a transwell system but maintained under a continuous flow. We refer to these slides as TripleB slides, incorporating three of the most important parameters, i.e., human brain endothelial cells, astrocytes and a laminar flow, to exemplify a valid and a functional BBB. The multi-layered microfluidic device was fabricated using three-dimensional resin printing (Objet Eden260VS, Stratasys). The TripleB slide consisted of four rigid bodies that were printed from the well-known MED610 resin material (Objet Eden260VS, Stratasys), as it is medically biocompatible and has reasonably good transparency. These four rigid bodies are shown in Figure 1a,c which provided the upper and lower fluidic channels for human endothelial cell growth and astrocyte growth, respectively. The channel cross section is a rounded rectangular shape with a channel width of 4 mm and height of 0.4 mm. The lower channel comprises input and output Luer-lock connectors with the same footprint as a microscope slide (25 × 75 mm). The upper channel also contains input and output Luer-locks to enable a connection and a pump controller to generate a flow. Part 1 and Part 2 of these upper and lower channels need to be joined together to ensure water-tightness, and this was achieved by using MED610 resin, which cures under UV light, as a potting adhesive. The two parts were assembled and then cured in a UV lamp. This two-step process is essential because even though 3D printing allows for the generation of complex structures, the Objet printer uses a support material which needs to be cleaned out before use—if 3D printed in a single step it would be difficult to effectively clean out this support material at the corners of the Luer-lock connectors and very small geometries (<1 mm), so to reduce contamination from the support material, the closed upper and lower channels were formed using this two-step process. Additionally, the TripleB slide makes use of a novel removeable membrane assembly, shown in Figure 1b, which consists of a porous membrane with 3.0 µm pore size (Polyethylene terephthalate, Oxyphen, Lachen, Germany), which is bonded to spacer tape (Tesa, Norderstedt, Duitsland) for support and sandwiched between two silicone rubber gaskets (Rubbermagazijn, Zoetermeer, Nederland). The silicone rubber gasket has a thickness of 1 mm and a Shore hardness of 30 A. The geometries shown here form an opening from one side of the membrane to the other (endothelial cells to astrocytes, respectively), which has a surface area of 4 × 8 mm (W × L) or 0.32 cm^2^. This size was chosen as it is close to the culturable surface area of a standard 24-well transwell insert (0.33 cm^2^). These components of the removable membrane were shaped using a combination of mechanical die-cutting and punching. It is possible to laser cut the porous membrane and spacer tape (whereas with silicone rubber it is not), but to reduce contamination of the membrane from residue from the laser-cutting process, mechanical die-cutting and punching was used. These three sub-assemblies were combined together, as shown with (d) a render and (e) a photograph of the fabricated TripleB fluidic chip. A photograph of the novel transwell insert is shown in (f) which comprises the two rigid bodies (the flanged carrier and the cover with bayonet-type connection) and the removable membrane assembly.

**Figure 2:**
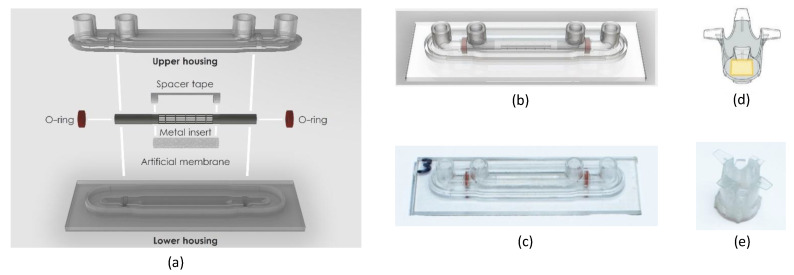
Demonstrations of the flexibility of prototyping devices with unconventional geometries by 3D-printing using a Polyjet printer, including (**a**) an exploded view of a 3D, vascular capillary-shaped BBB model based on Polyjet-printed rigid upper and lower housings to form two fluidic channels, containing a 1-mm diameter hollow cylinder which was micromachined as a scaffolding structure for the membrane layer, which is affixed to the scaffold with spacer tape. Mechanically punched silicone o-rings were used to seal the inner and outer channels from each other; (**b**) a render of the device, and (**c**) a photograph of the final prototyped device; (**d**) a CAD model of a novel transwell insert based on a dimensions of an insert for a standard 24-well transfer plate, where the yellow section is a removable 2 × 2 mm membrane with a 3D-printed frame, which was inserted into a 3D-printed flanged carrier; (**e**) a photograph of the prototyped novel 24-well transwell plate insert with removable membrane.

Another example of the flexibility of the 3D printing fabrication method is shown here in Figure 2, where a cylindrical geometry for a BB model was proposed and prototyped to demonstrate how a 2D porous membrane could be processed to form a complete cylinder, mimicking the form of a vascular capillary. Figure 2a shows the exploded view of the 3D cylindrical slide, which comprises rigid upper and lower housings that form two concentric fluidic channels and were 3D printed from MED610 resin material. The upper housing consists of the upper halves of the two fluidic channels and the Luer-lock connectors for fluid flow, and the lower housing consists of the lower halves of the fluidic channels and has the same footprint as the TripleB slide (25 × 75 mm). These rigid housings were bonded again using the same approach as the TripleB slide, with grooves for adding MED610 resin, which was separately cured under a UV lamp. The 3D cylindrical slide also contains a 1 mm diameter micromachined cylinder insert (Raytech, Bruge, Belgium) which acts as a rigid scaffolding structure for the porous membrane and was attached using spacer tape. Silicone rubber O-rings were used to seal the inner and outer channels, used for seeding human endothelial cells and astrocytes, respectively, and were mechanically punched from the same material used in the removable membrane. The cylindrical membrane assembly and silicone rubber O-rings were added to the upper and lower rigid housings, which were once again sealed using the same approach as the TripleB slide, by applying uncured MED610 resin with a syringe to the perimeter of the upper and lower housings, which were then separately cured under a UV lamp. A render of this assembly and a photograph of the prototyped device are shown in Figure 2b,c, respectively. Unlike the TripleB slide, this device does not have a removable membrane, so this model needs to be destructively disassembled if microscope imaging of the growth on the membrane is necessary. In addition to this 3D cylindrical slide geometry, a novel transwell insert with a removeable membrane was designed for a 24-well insert, as shown in Figure 2d, but this could only achieve a porous membrane with an active surface area of 2 × 2 mm or 0.04 cm^2^, shown here in yellow, which is significantly smaller than the active area of a standard 24-well transwell insert (diameter of 6.5 mm, and area of 0.33 cm^2^). The addition of the support frame for the porous membrane reduces the available surface area for the removeable membrane when designing around the dimensions of the standard 24-well plate. Therefore, a novel transwell insert for a standard 6-well plate was developed for the TripleB. Nonetheless, this novel 24-well insert was 3D printed using MED610 resin, shown in Figure 2e, to demonstrate that this 3D printing platform can produce small and precise functional prototypes.

**Figure 3:**
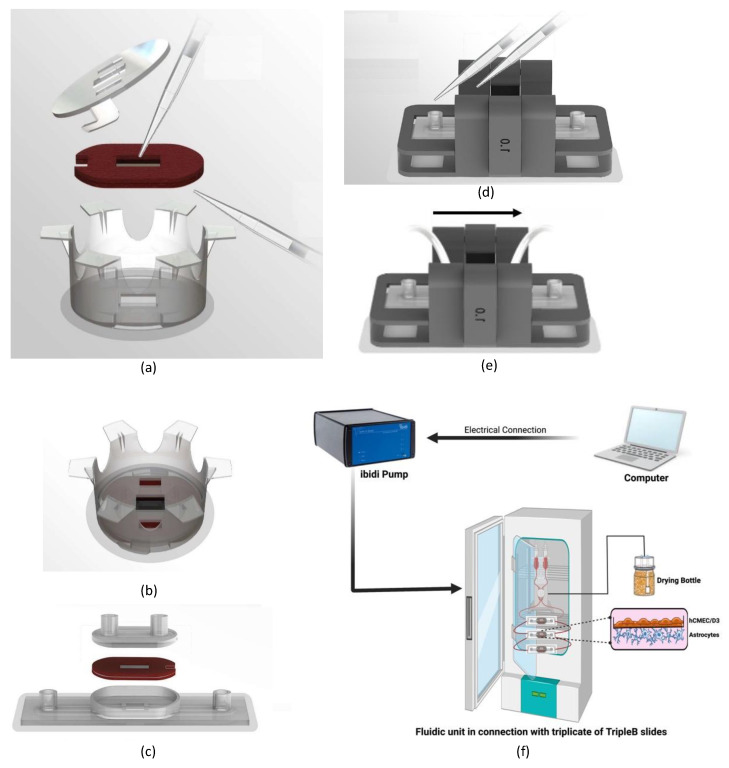
The clinical user flow of the TripleB model. Firstly (**a**) the red-colored removable membrane is seeded using pipettes with poly-L-lysine and endothelial cells on the upper half of the membrane and collogen and astrocyte cells on the lower half. The seeded membrane is then positioned inside the novel 6-well transwell insert, in this case the flanged carrier component, which is then secured in place with a 3D-printed cover with bayonet-type connections to keep the membrane barrier sealed. Once assembled (**b**), this can be placed in a standard 6-well transwell plate with the required nutrients in the well and on the inside of the insert for the initial incubation. After the initial incubation, (**c**) the membrane can be removed from the novel transwell insert and placed in the 3D-printed lower channel housing of the TripleB, which is then covered with the upper channel housing. The assembled fluidic chip is then placed into a 3D-printed clamping device (**d**) to ensure the fluidic channels do not leak, and then growth media can be added via pipettes to Luer-locks inputs of the upper and lower channels, to support the growth of endothelial cells and astrocyte cells, respectfully. From there, (**e**) fluidic flow tubes from the Ibidi pump are connected to the input and output Luer-locks of the upper channel to induce a strain on the endothelial cells due to the flow circulating through the system (which is indicated in the direction of the arrow), shown in (**f**) where three TripleB models are connected in parallel and further incubated under flow conditions.

The workflow of using the TripleB fluidic slide is illustrated in Figure 3. Firstly (Figure 3a), the removable membrane was seeded using pipettes with poly-L-lysine and endothelial cells on the upper half of the membrane, and collogen and astrocyte cells on the lower half. The seeded membrane was then positioned inside the novel 6-well transwell insert, in this case the flanged carrier component. To keep the interface between the inner and outer faces of the novel transwell insert sealed, a 3D-printed cover with a bayonet-type connection was attached from the inside of the insert to compress the silicone rubber gaskets against the flanged carrier. Once assembled (see Figure 3b), this was placed in a standard 6-well transwell plate with the required nutrients in the well and on the inside of the insert for initial incubation. After the initial incubation, the membrane was then removed from the novel transwell insert and placed in the 3D-printed lower channel housing of the TripleB, as shown in Figure 3c, which was then covered with the upper channel housing. The assembled fluidic chip was then placed into a custom-designed clamping device (Figure 3d), to ensure the fluidic channels do not leak. The clamp system consists of an 8 mm thick acrylic plate (Simplyplastics, Colchester, UK) that covers the upper channel assembly and 3D-printed parts generated using fused deposition modeling (FDM). There is a 1.0 mm overlap area between the clamp and the acrylic plate. This overlap causes the layers to be pressed firmly with sufficient force to prevent leakage but not distort the shape of the porous membrane. Then, growth media can be added via pipettes to Luer-locks inputs of the upper and lower channels, to support the growth of endothelial cells and astrocyte cells, respectfully. From there, fluidic flow tubes from the Ibidi pump (ibidi GmBH, Munich, Germany), were connected to the input and output Luer-locks of the upper channel, shown in Figure 3e, to replicate and maintain an artificial blood flow, thereby inducing a strain across the endothelial cells. The pump was connected in parallel with three TripleB slides which were placed in a humidified 5% CO_2_ atmosphere at 37 °C, shown in Figure 3f. The whole system maintains a unidirectional flow. The flow was maintained at a shear stress level ranging between 4–15 dyne/cm^2^ and the experiment ran for infinite cycles until the end of the experiment. The lower channel of the TripleB was not under flow and was capped with 3D printed plugs. This channel served as the abluminal compartment of the brain which could be used to collect the transmigrated drugs or cells.

### 2.2. Flow Simulation Using Computational Fluid Dynamics

In order to simulate the direction of flow inside the TripleB slides connected to the pump, computational fluid dynamics were performed. The computer-generated simulations of the fluidics for the design of the 3D slides ensured if the 3D slides are effectively compatible with the pump and indicated the type of flow generated inside these slides. For this we used the software SOLIDWORKS to generate flow simulations in the bottom chamber of the 3D slides. Three different design points were chosen, i.e., the middle potion of the membrane and the two extreme ends of the slides. An initial computational fluid dynamics visualization established the average velocity inside the slides, for the flow through the ‘tube’ system (inlet velocity of the fluid: 0.5 mm/s).

### 2.3. Cell Culture of BBB Models

Before culturing cells under dynamic flow, human primary astrocytes (Sanbio, Uden, The Netherlands) were seeded at a density of 15,000 cells/cm^2^ on the poly-L-lysine-coated samples (Sigma-Aldrich BVBA, Overijse, Belgium) outside of the TripleB membrane first and were allowed to adhere for 2 h. For this, the membrane was placed in the custom transwell (Figure 1a) and subsequently transferred into a 6-well plate (Greiner Bio-one, Vilvoorde, Belgium) filled with endothelial cell growth medium (EGM)-2-MV medium (Lonza, Verviers, Belgium) supplemented with 2.5% fetal bovine serum (FBS; Thermo Fisher Scientific, Erembodegem, Belgium). Next, human cerebral microvascular endothelial cells (hCMEC/D3; Tébu-bio, Le Perray-en-Yvelines, France) were seeded onto the collagen-coated membrane (Thermo Fisher Scientific, Erembodegem, Belgium) inside at a density of 25,000 cells/cm^2^. The cells were allowed to adhere to the membrane of TripleB slides for 1 h in the 6-well plate in a humidified 5% CO_2_ atmosphere at 37 °C.

After successful seeding, the membrane was placed in the engineered microfluidic slide, clamped with the clamping system and was then attached to the fluidic unit of the pump. Cells were maintained under shear stress levels of 4–15 dyn/cm^2^, which are considered comparable to those reported in brain capillaries in vivo [37,38,39,40,41]. At the start of the experiment, the shear stress level was maintained at 4 dyn/cm^2^, as a greater shear stress led to the detachment of the cells from the membrane. Following 2 h, the shear stress level was increased to 6 dyn/cm^2^ and at day 2 the shear stress was further increased to 8–15 dyn/cm^2^, mimicking the capillary-like shear stress values. A higher value of shear stress resulted in the disruption of the cells and hence these values was not used for these experiments [42]. The flow rate and pressure of the system varied from 3.7 mL/min and 18.2 mbar (for 4 dyn/cm^2^) to 25.11 mL/min and 38.6 mbar, respectively (for 15 dyn/cm^2^).

As a control, the static in vitro BBB model was constructed as described previously [43,44]. In brief, human primary astrocytes (Sanbio) were seeded at a density of 15,000 cells/cm^2^ on the poly-L-lysine-coated samples outside of a 24-well transwell with a 3.0 µm pore size (Polyethylene terephthalate, Greiner Bio-one) and were allowed to adhere for 2 h. The transwell insert was transferred into a 24-well plate filled with EGM-2-MV medium (Lonza) supplemented with 2.5% FBS (Thermo Fisher Scientific). Next, hCMEC/D3 cells (Tébu-bio) were seeded onto the collagen-coated membrane inside at a density of 25,000 cells/cm^2^. An equal number of cells was cultured in the static and dynamic BBB, as the culture surface of the membrane in the static BBB model was 33.6 mm^2^, which was similar to that of the porous membrane in the dynamic BBB system (with a culture surface area of 32 mm^2^ on the membrane).

Two days after initiating the coculture, the growth medium was replaced by endothelial basal medium (EBM-2)-plus, consisting of EBM-2 (Lonza) supplemented with 1.4 μM hydrocortisone (Sigma-Aldrich BVBA, Overijse, Belgium), 1 ng/mL basic fibroblast growth factor (bFGF, Thermo Fisher Scientific, Paisley, UK), 10 μg/mL gentamicin, 1 μg/mL amphotericin-B and 2.5% FBS, in both the static control BBB and the dynamic BBB model. For replacing the medium from the dynamic BBB, the fluidic units were taken out of the incubator and the experiment was paused for a very brief interval. The medium was pipetted out of the syringes and new medium was added using 10 mL pipettes inside the laminar flow. The fluidic unit was then again attached to the pump system and the experiment was resumed. The EBM-2-plus was replenished every other day.

All cell cultures were maintained in a humidified 5% CO_2_ atmosphere at 37 °C.

### 2.4. TEER Measurement

To check the efficiency of the BBB formation, the transendothelial electrical resistance (TEER) was measured at different time points after establishing the BBB coculture, starting from 72 h post-culture of BBB. TEER was determined using the EVOM-2 voltohmmeter with STX electrodes (World Precision Instruments, Hitchin, Hertfordshire, United Kingdom). Measurements were performed in duplicate, and the final mean TEER value is expressed in Ω cm^2^. Background TEER values, i.e., mean TEER across an empty collagen-coated insert, were subtracted from the mean TEER value recorded across BBB cocultures. For the dynamic model, the membrane was removed and placed in the specially designed custom transwell at each time point of measurement and then placed back in the TripleB slides for the further culturing of cells.

### 2.5. FITC–Dextran Permeability Assay

To assess BBB permeability, 100 μg/mL 4 kDa FITC–dextran solution (Merck-Sigma-Aldrich, Overijse, Belgium) was added to the upper compartment of the insert in the static BBB model. For the dynamic BBB model, the membrane was first removed from the TripleB slide and transferred to the custom 6-well transwell which was placed in a 6-well plate. Subsequently, the same concentration of FITC–dextran solution was added to the upper compartment of the custom built transwell (100 μg/mL). The fluorescence recovery in the lower compartment was measured after 60, 120, and 180 min using a Victor3 multilabel fluorometer in both models. As a positive control, 100 μg/mL FITC–dextran was directly added into the lower chamber of the static and the dynamic BBB model and was compared with a cell-free insert. The negative control consisted of medium only. The apparent permeability coefficient, Papp, was evaluated according to the following equation:

$$\mathrm{Papp}\;(\frac{\mathrm{cm}}{s})=\frac{\mathrm{dQ}}{\mathrm{dt}}\;\times \;\frac{1}{A\;\times \;C_{0}}$$

where dQ/dt is the amount of FITC–dextran present in the basal compartment as a function of time (nmol/s), A is the surface area of the membrane (0.33 cm^2^ for static BBB and 0.32 cm^2^ for TripleB) and C_0_ is the original concentration of FITC–dextran added in the upper chamber at the start of the experiment (nmol/s) [45,46,47,48].

### 2.6. Immunoflourescence Imaging

For immunofluorescence imaging, the BBB monoculture comprising hCMEC/D3 alone was cultured on the membranes of the static and the dynamic model. At days 3, 5 and 8, the membranes were cut and placed on a microscopic slide and subsequently fixed, blocked, and permeabilized using 4% paraformaldehyde (Sigma), and 0.01 M PBS (pH 7.4) supplemented with 0.05% thimerosal (Sigma), 10% normal horse serum, and 1% Triton X-100 (Sigma), respectively. Next, the cells were stained overnight using the following primary antibodies: a mouse anti-human−1 antibody (1/100) (BD Pharmingen, Erembodegem, Belgium). Next, cells were stained with a secondary fluorescein isothiocyanate (FITC)-labeled donkey anti-mouse antibody (1/100) (Jackson ImmunoResearch, Newmarket, UK) for 2 h at room temperature. Finally, cells were counterstained with 4′,6-diamidino-2-phenylindole (DAPI) (Sigma), and the membrane containing the cells was mounted in citifluor (Citifluor Ltd., London, UK) following its careful removal from the silicon casket of TripleB membranes and the transwell insert, and was subsequently stored at 4 °C. The images were taken using an UltraVIEW confocal microscope system (Perkin Elmer, Waltham, MA, USA). The analysis of the images was performed using the ImageJ software (National Institutes of Health, Bethesda, MD, USA).

### 2.7. Flow Cytometric Analysis

To assess the phenotype of the endothelial cells, the astrocytes were first removed mechanically from the TripleB membrane underside and the hCMEC/D3 cells cultured on the upper side of the membrane were detached with trypsin–EDTA (Thermo Fischer Scientific, Paisley, UK) and washed. The phenotype of the hCMEC/D3 cells was characterized using the following fluorochrome-labeled mouse anti-human monoclonal antibodies: anti-CD31–FITC (BD Pharmingen, Erembodegem, Belgium), anti-CD54–phycoerythrin (PE; BD Pharmingen), anti-CD45–peridinin chlorophyll (PerCP, BD Biosciences, Erembodegem, Belgium). Isotype-matched control monoclonal antibodies were used to determine non-specific background staining. For analytical flow cytometry, at least 104 events were analyzed using a Beckman Coulter CytoFLEX flow cytometer (Analis, Namur, Belgium). All results were analyzed using FlowJo software (Tree Star, Ashland, Ohio USA). For the analysis, the cells were directly plotted in the forward and side scatter format to gate the leukocytes from which the single cells were obtained next. These were then used to study the percentage of positive cells for the specific fluorophores of interest including their respective isotypes. Propidium iodide (PI) (Invitrogen™, Thermo Fischer) staining was used to check the viability of the cells.

### 2.8. RNA Isolation and Quantitative Real-Time Polymerase Chain Reaction (qPCR)

For the analysis of tight junction molecule expression, total RNA from hCMEC/D3 endothelial cells, which were cocultured with astrocytes in the dynamic and static in vitro BBB model, was isolated. For this, cells cultured on the membrane were detached with trypsin–EDTA. Before cell lysis, astrocytes were removed mechanically from the insert underside. Total RNA was isolated using the RNeasy microkit (Qiagen, Antwerp, Belgium). The RNA concentration was determined by measuring absorbance at 260 nm using a Nanodrop spectrophotometer (Wilmington, DE, USA). Reverse transcription of the obtained RNA into cDNA was performed using the iScript™ Advanced cDNA Synthesis Kit (Bio-Rad, Temse, Belgium). Subsequently, the SYBR^®^ Green technology was used for relative mRNA quantification by qPCR in a CFX96 C1000 thermal cycler (Bio-Rad). qPCR reactions were conducted at 95 °C for 2 min, followed by 40 cycles at 95 °C for 5 s and at 60 °C for 30 s. All primer sets, including glucose transporter 1 (SLC2A1), zonulin 1 (ZO-1), occludin 1 (OCLDN1) and claudin 5 (CLDN5), were obtained from Bio-Rad. qPCR was performed in triplicate and the resulting mRNA levels were normalized to levels of the reference gene glyceraldehyde 3-phosphate dehydrogenase (GAPDH). A melt curve analysis was performed to confirm the specificity of the amplified product. Bio-Rad CFX manager v3.1 was used for data processing and analysis.

### 2.9. Migration Assay

Migration assays were performed between days 5 and 6 of culture, when the co-cultures established functional barrier properties. Peripheral blood from healthy donors was obtained from buffy coats provided by the Red Cross donor center (Red Cross,; Flanders, Mechelen, Belgium). Peripheral blood mononuclear cells (PBMC) were isolated by density gradient centrifugation (Ficoll Pacque PLUS, GE Healthcare, Amsterdam, The Netherlands). The transmigration of the isolated PBMCs was then studied across steady-state and dynamic BBB cocultures. On the day of migration, the static BBB cocultures were transferred to a new well plate. For the dynamic BBB, the membranes were transferred into the custom 6-well transwell (Figure 1f) when the cells were sufficiently confluent which was then placed into a 6-well plate. Following this, 5 × 10^5^ PBMCs resuspended in IMDM supplemented with 1% human AB serum were added to the upper compartment of both the transwell and the dynamic BBB model. As a positive control, 5 × 10^5^ PBMCs were added directly to the lower compartment of both static and dynamic BBB. The negative control contained no added cells but only IMDM medium supplemented with 1% human AB serum to the basolateral compartment. PBMCs were subsequently allowed to migrate for 20–24 h in the assays using the in vitro BBB models. At the indicated time points, the migrated cells were collected from the basolateral compartment, while non-migrating cells were recovered from the upper compartment from the dynamic and static BBB models. Recovered cells were counted using a Neubauer counting chamber (Marienfeld, Germany). The percentage of migration was calculated as follows: [(# migrated cells from the experimental samples-# migrated cells from negative controls)/# migrated cells from positive controls] ∗ 100%.

### 2.10. Statistical Analyses

Data were analyzed using Graphpad Prism software version 5.01 (Graphpad, San Diego, CA, USA). For the comparison of 2 groups, a Mann–Whitney U test was used as the data set was considered non-parametric. When comparing 3 groups or more, statistical analysis was performed using Kruskal–Wallis one-way analysis of variance. Statistical significance was considered when *p* ≤ 0.05.

## 3. Results

### 3.1. Laminar Flow Is Maintained in TripleB Slides

**Figure 4:**
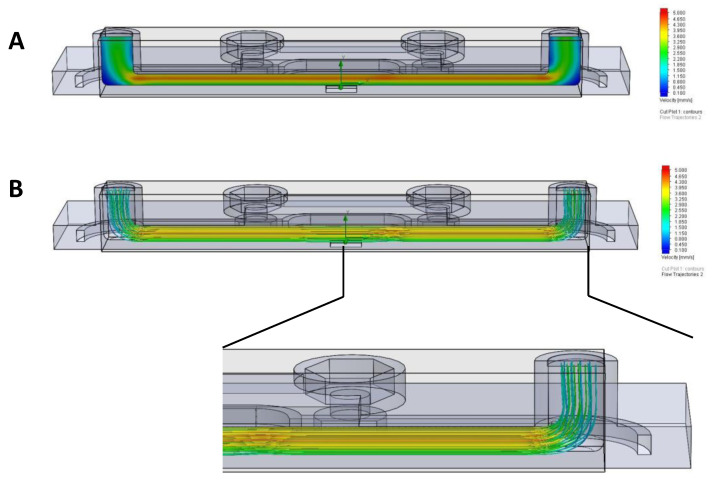
The 3D TripleB slides demonstrate the presence of continuous laminar flow. To model the flow inside of the microfluidic slide, the CFDs for the 3D designed slide were generated, which shows (**A**) the range of velocities as measured from 3 different positions in the bottom chamber of the slide (**B**) and the presence of unidirectional laminar flow in the TripleB slides.

First, to verify that the flow in the designed TripleB slides was laminar, we performed flow simulations in the bottom chamber of the slide. To determine the average velocity inside the slide, three distinctive design points were chosen, i.e., the two extremes of the slides adjacent to the inlet and outlet ports and the mid membrane portion (Figure 2). Starting with an inlet velocity of 1.5 mm/s (comparable to the average velocity of a brain capillary), the maximum velocity as measured in the center of the membrane ranged between 4.3 mm/s and 5 mm/s. Similarly, the maximum velocity measured in the corners of the microfluidic slide was around 0.1 mm/s. The initial parameter of environment pressure was chosen to be 101,325 Pascal. From this, we established that if the velocity across the membrane should be 1.5 mm/s (comparable to the average velocity of a brain capillary) then the inlet velocity must be reduced to 0.5 mm/s (Figure 4A). Using the fluid velocity at the center of the slide, fluid viscosity and the geometry, the Reynolds number of the TripleB slide was calculated to be *Re_tb_* = 88, which is significantly lower than the limit for laminar flow, *Re_L_* < 2300. This conclusion was confirmed through an initial computational fluid dynamics (CFD) visualization, which exhibited that there was no sign of turbulent flow in the TripleB slides. Each line represents the flow of a “particle”. If there was a presence of turbulent flow in the slide, these lines would curl and bend, which was not the case (Figure 4B and inset). The detailed inset shows slight deviation of flow across the boundary where the membrane finishes and the 3D-printed geometry begins, but this is only minor.

### 3.2. The Endothelial Cell Layer of the BBB Aligns with the Direction of Flow in the Dynamic In Vitro Model Resulting in Pronounced Cell-to-Cell Tight Junction Formation

**Figure 5:**
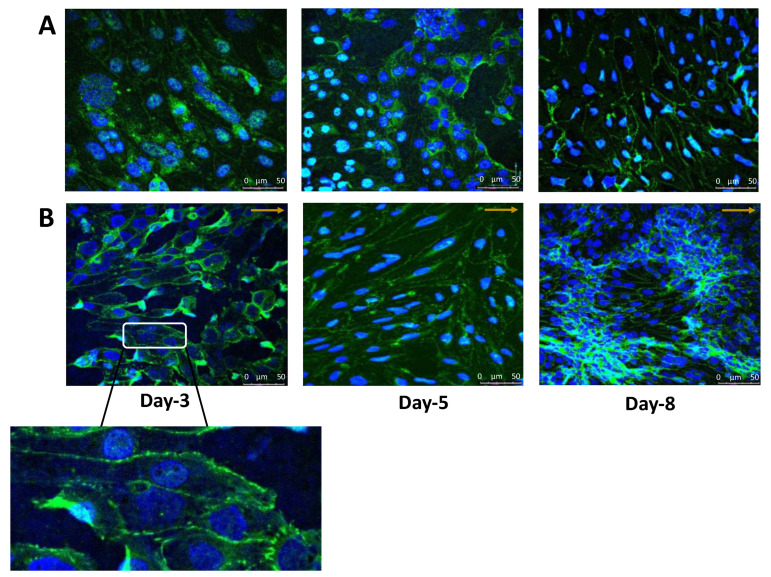
Endothelial cells grow in the direction of the laminar flow in dynamic BBB model. BBB was cultured under static conditions (**A**) or under a laminar flow of 4–15 dyn/cm^2^ (**B**) for 8 days Confocal images obtained following staining of the hCMEC/D3 cells for DAPI (blue), and ZO-1 (green) merged when grown on collagen-coated microporous membranes. Arrows indicate the direction of the flow. Images were taken using an UltraVIEW confocal microscope.

It was previously demonstrated by others [36,40,49,50,51] that the most profound alteration of endothelial cells in response to shear stress is the alignment of the cells in the direction of the flow. We therefore investigated how the morphology of the hCMEC/D3 changed in response to unidirectional laminar flow for 8 days. Under static conditions, the cellular morphology was indiscriminately organized with a discontinuous irregular pattern (Figure 5A). However, under shear stress, linear realignment of the hCMEC/D3 was particularly visible following day 3 and day 5 of continuous shear stress exposure and was largely localized to the intercellular junctions of cells with limited discontinuous tight junction formation (Figure 5B). Indeed, the expression of the tight junction protein ZO-1 was assessed on membranes maintained in either static media or exposed to shear stress. Under static conditions, limited cell-to-cell ZO-1 formation is evident (Figure 5A). When the capillary like shear stress levels of 4-15 dyn/cm^2^ were applied, cellular reorganization was conspicuous, with cell-to-cell ZO-1 protein formation and an elongated endothelial cell formation. However, on day 8 the endothelial cells started to form clusters and grow on top of each other in the form of cell layers, which was observed in the TripleB-cultured slides. This resulted in the loss of the linear alignment of the cells as previously observed at day 3 of culture. Meanwhile cells were gaining a better shape in the static BBB cultured on a transwell at day 8. This reduced expression of ZO-1 could be correlated with the increased proliferation of cells and/or transformation [52,53,54,55]. For example, in highly proliferative brain microvascular endothelial cells that from human brain tumors, ZO-1 levels are typically low [53]. Translocation of ZO-1 to the cytoplasm was, however, evident in both static and dynamic conditions, albeit being most pronounced at day 8 under laminar flow. Overall, these results are indicative of an in vivo-like morphology for the human brain ECs cultured on the TripleB, consequently validating structural requirements for BBB formation. This further suggests that the BBB formed in TripleB slides acquired a higher structural and functional integrity of the barrier and hence the dynamic interface between the peripheral circulation and the central nervous system is much stronger in these slides.

### 3.3. A Significantly More Stringent and Impermeable Barrier Was Formed in the Dynamic TripleB Model

**Figure 6:**
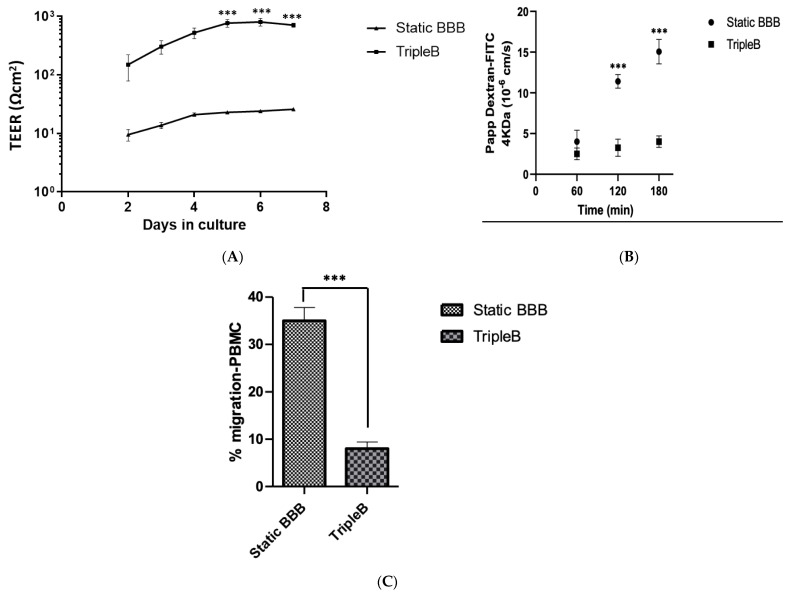
Highly rigorous blood–brain barrier formation takes place when the cells are cultured in the presence of flow. TEER was measured following the growth of hCMEC/D3 and astrocytes on permeable membranes (**A**) under static and dynamic (4–15 dyn/cm^2^) conditions. The TEERs of hCMEC/D3 were measured when grown on permeable inserts in the absence and presence of shear stress. Steady-state TEER values were typically reached in the static BBB by day 4 of culture and were attained by the TripleB slides by day 5. (n = 6). Permeability to the tracer molecule FITC–dextran; (**B**) a significant decrease in the apparent permeability to FITC–dextran was induced in the BBB cultured in dynamic flow conditions as compared to the static BBBs (n = 4) (**C**) The ability of immune cells to cross the BBB was significantly downregulated under dynamic flow conditions. Migratory capacity of PBMCs was evaluated across a static and a dynamic BBB. Significantly lower numbers of PBMCs were recovered from the TripleB culture as compared to the amount of cells harvested from a static culture of BBB. (n = 4, *** *p* ≤ 0.001).

To investigate the formation of a high resistance barrier, the TEER values were determined in static conditions and under dynamic shear stress at incremental time points. At day 5 post seeding, the TripleB model demonstrated a more than 25-fold higher TEER (869 ± 10.75 Ω cm^2^) as compared to the static BBB (32 ± 3.43 Ω cm^2^; *p* < 0.001), which was maintained through to day 8 post seeding (Figure 6A).

Next, we tested the permeability of the BBBs grown under static and dynamic culture using a FITC–dextran dye. The passage of FITC–dextran across a well confluent BBB was checked between day 5 to day 6 of cultures in both the systems. The TripleB membrane was placed in the custom built 6-well transwell. The fluorescent tracer was dissolved in serum-free media and added in the upper compartment of the transwell. Similarly, the dye was added in the top compartment of the static BBB and was detected from the abluminal chamber of both cultures at three different time points. A significantly reduced fluorescence (*p* < 0.001) was observed in the dynamic BBB when compared at 2 h and 3 h after adding the FITC–dextran to the static BBB cultures (Figure 6B), indicative of a less permissive membrane.

Besides the use of FITC–dextran to assess the permeability of the BBB, we investigated the permeability of the BBB for the migration of immune cells as a measure of the functional impact of a rigid barrier. For this, we determined the migratory potential of PBMCs towards a chemokine gradient across a well-formed dynamic in vitro BBB as compared to a static BBB model. Migration assays were performed between days 5 and 6 of culture, when the co-cultures established functional barrier properties. PBMCs were added to the circulating top channel of the slides cultured under laminar flow along with the luminal compartment of the BBB cultured in the static model and were allowed to migrate in a chemokine-dependent manner. After 20–22 h, cells were collected from the bottom channels of both BBB models. We observed 35.30 ± 2.67% of PBMCs migrating across the static BBB model, whereas the more rigid barrier formation under laminar flow resulted in a significantly lower percentage of migrating PBMCs across the dynamic TripleB model (7.5 ± 2.05%; *p* < 0.001) (Figure 6C). No significant difference was observed in the viability of the migrating PBMCs between the static (77.2 ± 2.35%) and the dynamic model (73.81 ± 3.14%).

Altogether, our results are indicative of highly rigorous barrier formation in the TripleB slides, showing that this type of culture system is highly advantageous over the BBBs cultured in the static environment.

### 3.4. Upregulated Expression of Different Tight Junction Proteins as Well as of the Hallmark BBB Protein, Pg-p, Was Found in the Dynamic TripleB Model

**Figure 7:**
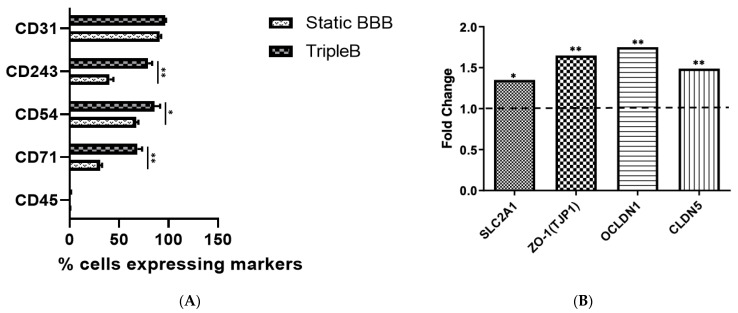
TripleB cultured barriers demonstrate a much higher expression of P-glycoprotein along with other endothelial-cell-specific markers when compared to transwell-cultured barriers. Phenotypic differences in the static and dynamic cultured BBB (**A**) BBB cultured in the TripleB slides showed a significantly higher expression of EC-specific markers compared with the static in vitro BBB. (**B**) mRNA encoding the tight junction proteins SLC2A1, TJP1, OCLDN1 and CLDN5 was found to be significantly upregulated in TripleB cultures as compared to the static BBB cultures (n = 4, * *p* ≤ 0.05; ** *p* ≤ 0.01).

Pg-p restricts or prevents entry to the brain for a wide variety of small lipophilic drugs, which presents a significant hurdle to the treatment of various CNS diseases. Hence, it is mandatory that a high-throughput and low-cost alternative for excessive animal testing demonstrates Pg-p expression. However, it was previously demonstrated that wild-type hCMEC/D3 cells exhibit a low expression of Pg-p and low junctional tightness under routine culture conditions [56]. For this, we studied the percentage of positive cells expressing P-glycoprotein (CD243), PECAM-1 (CD31), ICAM-1 (CD54), transferrin receptor (CD71) and CD45 in the static BBB model as well as the dynamic TripleB model using flow cytometry. We found a significantly higher phenotypic expression of CD243 (*p* < 0.01), CD54 (*p* < 0.05) and CD71 (*p* < 0.01) in the dynamic TripleB model when compared to the static BBB model (Figure 7A). No significant difference was observed in the expression of CD31 and CD45. Furthermore, mRNA encoding tight junction proteins, including claudin-5, ocludin-1 (OCLDN1), Zonulin-1 protein (ZO-1) and glucose transporter-1 (SLC2A1), was found to be significantly upregulated in the dynamic in vitro BBB when compared to the static in vitro BBB (Figure 7B).

These results point out that the TripleB cultured barrier is superior in mimicking the in vivo characteristics of the BBB when compared to the static cultured BBB, especially given the upregulated phenotypic expression of the hallmark BBB molecule, Pg-p.

## 4. Discussion and Conclusions

A reproducible in vitro model of the BBB that incorporates all features of an in-situ BBB [57,58,59] and that can be used to study the drug permeability as well as the migration of various immune cells would be a breakthrough in the biotechnological industry. The various functional and structural properties of an in-situ BBB mainly include the expression of BBB-specific markers, exposure to luminal flow membrane, inductive influence from glia, presence of tight junction proteins, a high TEER expression, low K+ permeability and stereoselective transport of glucose and amino acids [57]. In this study, we designed a novel dynamic in vitro microfluidic BBB utilizing an immortalized human brain endothelial cell line in combination with primary human astrocytes cultured under continuous laminar flow. While conflicting results have been reported regarding the effect of astrocyte coculturing on endothelial cells, it has been suggested that the proximity of the astrocytes and endothelial cells may be crucial while developing an in vitro BBB model [60,61,62]. Cell-cell contact-dependent mechanisms aid in the induction of BBB properties in endothelial cells. For instance, the most significant reductions in paracellular permeability were seen when astrocytes were grown on the basolateral side of the filter or on the plastic well plate surface in the same transwell as the brain endothelial cell lines. Moreover, it is thought that the model in which cells are grown on the bottom of the transwell-permeable support may lead to tighter junctions due to the ability of the astrocytic endfeet to migrate through the pores of the filter and interact with the BMECs through direct contact [60,63,64,65,66]. In addition, astrocytes secrete a range of soluble mediators known to affect the phenotype and functioning of BBB endothelial cells. Based on these findings, we applied here a similar model of culturing astrocytes and endothelial cells on opposing sides of a permeable membrane being more physiologically relevant due to the symbiotic signaling and differentiation that can occur when both cell types are grown in the same culture. 

We demonstrated that under continuous presence of shear stress, a tangential force generated by the flow of blood across their apical surfaces, a clear alignment of the cells towards the direction of flow was discernible, in agreement with observations by others [67]. It has been postulated that the endothelial cell alignment under laminar flow is an important atheroprotective, adaptive process, as evidenced by the dependence of endothelial cell signals, cell shape and cytoskeleton of the direction of flow. Moreover, endothelial cells demonstrate more pronounced and elongated endothelial cell shape and tight junction formation of Zonulin-1 after 3 days of exposure to shear stress under continuous flow. The higher fluorescence expression of Zonulin-1 was observed from the TripleB cultures under flow when compared to the static brain microvascular endothelial cells following 3 to 5 days of culture under continuous laminar flow. This observation was confirmed by RNA expression levels of ZO-1 (TJ1) demonstrating upregulated expression of this tight junction protein following exposure to laminar shear stress. Zonulin-1 being a peripheral protein localizing at junctional sites [14] is also found to be present in cytoplasm and nucleus of epithelial and endothelial cells which could be a direct effect of cell proliferation [53]. ZO-1 and ZO-2 are primarily present in cell nuclei of these cells but become re-distributed to the plasma membrane as soon as cells reach confluence [53]. With increased proliferation and/or transformation of cells a reduced expression of zonulin is observed with which is also correlated with its cytoplasmic or nuclear translocation [52,68,69,70]. ZO-1 further perturbs gene expression by sequestering the Y-box transcription factor ZONAB (ZO-1 associated nucleic acid binding protein) to the cytoplasm [55]. This suggests that nuclear or cytoplasmic accumulation of zonulin is a general response of epithelial and endothelial cells to environmental or mechanical stress. Furthermore, decreased expression of ZO-1 lead the reorganization of BBB-actin protein, which can also lead to an increase in BBB permeability [52,71]. Interestingly, we observed translocation of zonulin-1 into the cytoplasm of the endothelial cells at day-8 in the dynamic TripleB membranes which could be an effect of increased cell proliferation of the cells.

Tight junction protein complexes provide a mechanical means to seal the paracellular pathways between adjacent endothelial cells and hence shield the brain from unwanted and potentially harmful substances [10]. Besides ZO-1 expression, also RNA levels of genes encoding for a variety of other tight junctional proteins, including claudin and occludin, are increased in our dynamic Triple B model. This increase in the expression of tight junction proteins insinuates that a much secure barrier formation takes place in the TripleB slides restricting the passage of pathogens, the diffusion of solutes in the blood, and large or hydrophilic molecules into the cerebrospinal fluid [14,72,73,74]. This also results in a well-maintained CNS homeostasis in this model resulting in a much healthier BBB formation [75,76]. These findings are in agreement with others demonstrating a correlation between the expression of tight junction markers and shear stress conditions [36,42]. Although, we have only used RT-qPCR tests to study the expression of other tight junction proteins than immunofluorescence which could tell more about the precise location of proteins. This was based on various previous reports stating that RT- PCR is more sensitive with higher specificity than immunofluorescence assay (IFA) for detection of various proteins [77,78,79,80]. In addition to tight junction protein upregulation, we also found a significant increase in the protein expression levels of endothelial cell-specific markers, such as platelet endothelial cell adhesion molecule-1 (PECAM-1 or CD31) and intercellular adhesion molecule-1 (ICAM-1 or CD54). The stable expression of these molecules is known to play a critical role in the maintenance of human vascular endothelial barrier function and properties [81,82,83].

Since it is accepted that shear stress affects tight junction formation and the expression of junction-related proteins [84,85], we measured the transendothelial electrical resistance (TEER) across the BBB, an index of the endothelial ‘tightness’ of the monolayer [86]. Previous studies have established that the in vivo TEER levels are typically greater than 1000 Ω cm^2^ [14]. While TEER values across human BMECs cannot be easily measured in vivo, TEER values across rat and frog brain Ecs have been measured in the range of 1200–1900 Ω cm^2^ [87,88]. In our hands, co-culture of endothelial cells and astrocytes in the TripleB slides under unremitting exposure to flow resulted in a TEER value of 869 ± 10.75 Ω cm^2^ which is over 25-fold higher than in static conditions. Recently, Elbakary and Badhan demonstrated TEER values of 448.1 Ω cm^2^ ± 11.3 Ω cm^2^ under flow in a dynamic perfusion-based BBB model [39]. In another study Hui Xu and colleagues reported a dynamic in vivo-like organotypic BBB model with TEER values up to 1298 ± 86 Ω cm^2^ [89]. 

While our results provide proof-of-principle that under the presence of in vivo-like flow a sufficiently tight barrier is formed with adequately high TEER in the dynamic BBB, we investigated next whether our TripleB model can efficiently discriminate the passage of substances. We demonstrate a significant drop in the permeability of FITC-dextran across the dynamic TripleB model as compared to the static in vitro BBB. Interestingly, the dynamic TripleB model was significantly less penetrable by immune cells as compared to the static in vitro BBB model. Indeed, the transmigration capacity of PBMCs across the dynamic TripleB model was 3.5-fold lower than the migration of PBMCs in the static model. This suggests a tighter barrier formation limiting increased immune cell migration, which is in line with previous findings by Cucullo and colleagues demonstrating low to almost no extravasation of circulating monocytes in a dynamic in vitro BBB (DIV-BBB) system maintained under flow, whereas flow cessation followed by reperfusion in the same system caused a biphasic opening of the BBB resulting in extravasation of immune cells [90]. Importantly, the size and design of the lower compartment in our TripleB device make it possible to collect enough migrated cells for further downstream analysis, such as transcriptomics profiling of migrated cells and systems biology. Indeed, this system can be used to study not only the transport of novel therapeutics (drugs and other molecules) across the BBB but also the increased infiltration of various immune cells in the BBB following inflammation. While our findings attest that the dynamic TripleB model developed by us represents a highly functional and stern barrier formation, one of the most important aspects of a healthy BBB is constraining the accumulation of potentially toxic substances into the brain. This is achieved by a very vital transporter present at the BBB, namely the multidrug resistance protein P-glycoprotein, encoded by MDR1/ABCB1 and belonging to the family of ATP-binding cassette transporters [25,91]. Pg-p works as a gatekeeper in the BBB and is essentially one of the most important constituents for robust barrier formation in the BBB. Extensive experiments with in vitro models and with knockout mice lacking blood-brain barrier Pg-p or other animal models treated with blockers of Pg-p have shown that Pg-p is responsible for the very poor penetration of many relatively large (>400 Da) hydrophobic drugs in the brain, by performing active back-transport of these drugs to the blood [26,30,92,93,94,95]. Hence, expression of Pg-p in a dynamic microfluidic in vitro model of BBB would differentiate the model with characteristic brain-derived features. While it is known that the presence of astrocytes increases the functional expression of Pg-p in the in vitro BBB model [96], we found that the exposure to flow additionally increases phenotypic expression of the efflux transporter Pg-p (CD243) in the TripleB cultured BBB when compared to the static in vitro BBB model, but we have not precisely checked the functional expression of Pg-p in the TripleB cultures in this study. In addition to Pg-p, other ABC efflux transporters such as members of the multidrug resistance protein (MRP) family and breast cancer resistance protein (BCRP) seem to contribute to BBB function. In particular, BCRP (ABCG2) is known to have beneficial effects while studying the role of drug deposition and contribute to BBB function [21,97,98]. Besides, we also observed that the expression of transferrin receptor (CD71) was significantly amplified in the TripleB system in comparison to the static system of BBB. The transferrin receptor (TfR) is known to be responsible for the transport of iron into the brain parenchyma to maintain iron homeostasis [99,100]. Also, glycocalyx-related genes and pathways are known to be affected by the cerebral blood flow [101] but we have not precisely checked for its expression in this study. 

Recent technological advancements have contributed to the development of various novel in vitro 3D models of the BBB. Indeed, current innovations in organ-on-a-chip (OACC) technology have provided the ability to recapitulate the microenvironment of the BBB. This technology allows for the recreation of tissue barriers in vitro with the added feature of establishing microfluidic circuits to perfuse modeled organs across an array of multiple, interconnected chips [102,103,104]. However, the initial preparation of the organ-on-a-chip BBB models and the injection of cells are still burdensome manual processes. These procedures are not only hard to operate, but also result in lost accuracy and consistency from batch to batch [105,106,107]. In this study, we have taken the first step to implement a microfluidics system on a cell culture model of the BBB. We have demonstrated the ease to incorporate BBB in-vitro models into the commercially available ibidi pump system without any unnecessary complications. In doing so, the TripleB model was able to reproduce all the key critical characteristics of the BBB as observed in situ. Especially the presence of P-glycoprotein, which is one of the most critical protein present across the BBB, in this device is one of the new and advantageous feature of this system. Nonetheless, our model remains a simplistic representation of the BBB. One of the most important factors missing from this model is the absence of pericytes. Previous reports have established that pericytes play a critical role in the integration of endothelial and astrocyte function at the neurovascular unit, and in the regulation of the BBB in vitro [108,109]. Hence, including pericytes into an in vitro model of the BBB could provide additional phenotypic advantages in mimicking the in vivo BBB. Ultimately, we envisage that the field will evolve to more complex organoid models of BBB holding much promise for advancing neuroscience research and outpacing in vivo models [104,110,111]. These systems have the capability to provide much data comparable to in vivo studies but one of the major limitations of current cerebral organoids (both human and mouse) is that they lack vasculature and blood circulation [105]. This imposes severe constraints on the maximum size that organoids can grow to, and the extent to which they can develop normally [89,111]. 

Recently, iPSC-derived BBB models have become popular due to its reprogramming and differentiation abilities. iPSCs circumvent the ethical issues comparing to animal models and embryonic stem cells [112,113,114,115,116]. Previous studies have reported the use of iPSCs and silicon nanomembrane platform for the design and study of in vitro BBB and immune cell interaction [117,118]. However, although there are several successful BBB-on-a-chip models derived from iPSCs, the cell culture protocols are not mature and have a high rate of failures [112]. Also, it has been previously reported that derivation of ECs with BBB properties from pluripotent stem cells can suffer from several limitations including the complexity of the differentiation process, the reproducibility of the system and the uncertainty in terms of stability [119,120]. 

We have not checked for the permeability levels of potassium (K+) ions and sucrose into the BBB. The BBB maintains a specific combination of specific ion channels and transporters, which keep the ionic composition optimal for neural and synaptic signaling functions [121]. K+ ion permeability has been widely studied in different BBB models since potassium homeostasis in known to control neuronal excitability but is also essential for the transmigration of macrophage across the BBB and is also involved in the modulation of BBB integrity and endothelial morphology [58,122,123]. Furthermore, sucrose in blood and brain has been routinely used as a quantitative measure of the in vivo blood-brain barrier (BBB) integrity [124]. In particular, radiolabeled [14C] sucrose has been extensively used for quantitative determination of BBB integrity in many in vitro [125,126,127], in situ brain perfusion [128,129,130,131], and in vivo [132,133,134] studies. This is because sucrose is a water-soluble molecule with no significant metabolism after parenteral injection, no binding to plasma or tissue proteins, and very low permeability (<10^−7^ cm/s) across the intact BBB.

In conclusion, in this study, we have demonstrated the impact of shear stress to an easy to isolate, user-friendly and highly reproducible BBB model derived from human brain cell line (hCMEC/D3) and human primary astrocytes co-cultured under continuous laminar flow. Microfluidic systems are recently gaining interests to be used as research tools for perfusion-based cell culture systems but remain rare because of their increased complications in handling. Here, we have concocted a basic and easy to use microfluidic device which can exploit the advantageous handling features of the transwell systems since most research groups working on barrier models are still using permeable-inserts (e.g., Transwell) systems for assessment of the impact of perfusion on the functional activity of barrier due to commercial availability, high-throughput potential, and ease of use. This system highly mimics the stable and meticulous in vivo BBB characteristics. Such a device has great potential in CNS diseases and drugs screening and is destined to bring technological innovation to human brain research, and mutatis mutandis other barrier systems such as the gut.

## References

[1] Deuschl G., Beghi E., Fazekas F., Varga T., Christoforidi K.A., Sipido E., Bassetti C.L., Vos T., Feigin V.L. (2020). The Burden of Neurological Diseases in Europe: An Analysis for the Global Burden of Disease Study 2017. Lancet Public Health.

[2] Feigin V.L., Vos T., Alahdab F., Amit A.M.L., Bärnighausen T.W., Beghi E., Beheshti M., Chavan P.P., Criqui M.H., Desai R. (2021). Burden of Neurological Disorders across the US from 1990-2017: A Global Burden of Disease Study. JAMA Neurol..

[3] Feigin V.L., Abajobir A.A., Abate K.H., Abd-Allah F., Abdulle A.M., Abera S.F., Abyu G.Y., Ahmed M.B., Aichour A.N., Aichour I. (2017). Global, Regional, and National Burden of Neurological Disorders during 1990–2015: A Systematic Analysis for the Global Burden of Disease Study 2015. Lancet Neurol..

[4] Chen Y., Dalwadi G., Benson H.A.E. (2004). Drug Delivery across the Blood-Brain Barrier. Curr. Drug Deliv..

[5] Mulvihill J.J.E., Cunnane E.M., Ross A.M., Duskey J.T., Tosi G., Grabrucker A.M. (2020). Drug Delivery across the Blood–Brain Barrier: Recent Advances in the Use of Nanocarriers. Nanomedicine.

[6] Abbott N.J., Patabendige A.A.K., Dolman D.E.M., Yusof S.R., Begley D.J. (2010). Structure and Function of the Blood--Brain Barrier. Neurobiol. Dis..

[7] Abbott N.J. (2005). Physiology of the Blood–Brain Barrier and Its Consequences for Drug Transport to the Brain. International Congress Series.

[8] Pandit R., Chen L., Götz J. (2020). The Blood-Brain Barrier: Physiology and Strategies for Drug Delivery. Adv. Drug Deliv. Rev..

[9] Daneman R., Prat A. (2015). The Blood–Brain Barrier. Cold Spring Harb. Perspect Biol..

[10] Haseloff R.F., Dithmer S., Winkler L., Wolburg H., Blasig I.E. (2015). Transmembrane Proteins of the Tight Junctions at the Blood–Brain Barrier: Structural and Functional Aspects. Seminars in Cell & Developmental Biology.

[11] Jiang X., Andjelkovic A.v., Zhu L., Yang T., Bennett M.V.L., Chen J., Keep R.F., Shi Y. (2018). Blood-Brain Barrier Dysfunction and Recovery after Ischemic Stroke. Prog. Neurobiol..

[12] Alavijeh M.S., Chishty M., Qaiser M.Z., Palmer A.M. (2005). Drug Metabolism and Pharmacokinetics, the Blood-Brain Barrier, and Central Nervous System Drug Discovery. NeuroRx.

[13] Cardoso F.L., Brites D., Brito M.A. (2010). Looking at the Blood–Brain Barrier: Molecular Anatomy and Possible Investigation Approaches. Brain Res. Rev..

[14] Luissint A.-C., Artus C., Glacial F., Ganeshamoorthy K., Couraud P.-O. (2012). Tight Junctions at the Blood Brain Barrier: Physiological Architecture and Disease-Associated Dysregulation. Fluids Barriers CNS.

[15] Nair M., Jayant R.D., Kaushik A., Sagar V. (2016). Getting into the Brain: Potential of Nanotechnology in the Management of NeuroAIDS. Adv. Drug Deliv. Rev..

[16] Gajdács M. (2019). The Concept of an Ideal Antibiotic: Implications for Drug Design. Molecules.

[17] Prinz M., Mildner A. (2011). Microglia in the CNS: Immigrants from Another World. Glia.

[18] Aday S., Cecchelli R., Hallier-Vanuxeem D., Dehouck M.P., Ferreira L. (2016). Stem Cell-Based Human Blood–Brain Barrier Models for Drug Discovery and Delivery. Trends Biotechnol..

[19] Ballabh P., Braun A., Nedergaard M. (2004). The Blood--Brain Barrier: An Overview: Structure, Regulation, and Clinical Implications. Neurobiol. Dis..

[20] Helms H.C., Abbott N.J., Burek M., Cecchelli R., Couraud P.-O., Deli M.A., Förster C., Galla H.J., Romero I.A., Shusta E.V. (2016). In Vitro Models of the Blood–Brain Barrier: An Overview of Commonly Used Brain Endothelial Cell Culture Models and Guidelines for Their Use. J. Cereb. Blood Flow Metab..

[21] Löscher W., Potschka H. (2005). Blood-Brain Barrier Active Efflux Transporters: ATP-Binding Cassette Gene Family. NeuroRx.

[22] Idriss H.T., Hannun Y.A., Boulpaep E., Basavappa S. (2000). Regulation of Volume-Activated Chloride Channels by P-Glycoprotein: Phosphorylation Has the Final Say!. J. Physiol..

[23] Gottesman M.M., Pastan I. (1993). Biochemistry of Multidrug Resistance Mediated by the Multidrug Transporter. Annu. Rev. Biochem..

[24] Virgintino D., Robertson D., Errede M., Benagiano V., Girolamo F., Maiorano E., Roncali L., Bertossi M. (2002). Expression of P-Glycoprotein in Human Cerebral Cortex Microvessels. J. Histochem. Cytochem..

[25] Schinkel A.H. (1999). P-Glycoprotein, a Gatekeeper in the Blood–Brain Barrier. Adv. Drug Deliv. Rev..

[26] Ramakrishnan P. (2003). The Role of P-Glycoprotein in the Blood-Brain Barrier. Einstein QJ Biol. Med..

[27] Van Assema D.M.E., Lubberink M., Boellaard R., Schuit R.C., Windhorst A.D., Scheltens P., Lammertsma A.A., van Berckel B.N.M. (2012). P-Glycoprotein Function at the Blood–Brain Barrier: Effects of Age and Gender. Mol. Imaging Biol..

[28] de Lange E.C.M., vd Berg D.J., Bellanti F., Voskuyl R.A., Syvänen S. (2018). P-Glycoprotein Protein Expression versus Functionality at the Blood-Brain Barrier Using Immunohistochemistry, Microdialysis and Mathematical Modeling. Eur. J. Pharm. Sci..

[29] Bauer M., Tournier N., Langer O. (2019). Imaging P-Glycoprotein Function at the Blood–Brain Barrier as a Determinant of the Variability in Response to Central Nervous System Drugs. Clin. Pharmacol. Ther..

[30] Fromm M.F. (2000). P-Glycoprotein: A Defense Mechanism Limiting Oral Bioavailability and CNS Accumulation of Drugs. Int. J. Clin. Pharmacol. Ther..

[31] Wilhelm I., Fazakas C., Krizbai I.A. (2011). In Vitro Models of the Blood-Brain Barrier. Acta Neurobiol. Exp..

[32] Gomes M.J., Mendes B., Martins S., Sarmento B. (2016). Cell-Based In Vitro Models for Studying Blood–Brain Barrier (BBB) Permeability. Concepts and Models for Drug Permeability Studies.

[33] Lecuyer M.-A., Kebir H., Prat A. (2016). Glial Influences on BBB Functions and Molecular Players in Immune Cell Trafficking. Biochim. Biophys. Acta (BBA)-Mol. Basis Dis..

[34] Abbott N.J. (2013). Blood–Brain Barrier Structure and Function and the Challenges for CNS Drug Delivery. J. Inherit. Metab. Dis..

[35] Srinivasan B., Kolli A.R., Esch M.B., Abaci H.E., Shuler M.L., Hickman J.J. (2015). TEER Measurement Techniques for In Vitro Barrier Model Systems. J. Lab. Autom..

[36] Cucullo L., Hossain M., Puvenna V., Marchi N., Janigro D. (2011). The Role of Shear Stress in Blood-Brain Barrier Endothelial Physiology. BMC Neurosci..

[37] Buchanan C.F., Verbridge S.S., Vlachos P.P., Rylander M.N. (2014). Flow Shear Stress Regulates Endothelial Barrier Function and Expression of Angiogenic Factors in a 3D Microfluidic Tumor Vascular Model. Cell Adh. Migr..

[38] Wong A., Ye M., Levy A., Rothstein J., Bergles D., Searson P.C. (2013). The Blood-Brain Barrier: An Engineering Perspective. Front. Neuroeng..

[39] Elbakary B., Badhan R.K.S. (2020). A Dynamic Perfusion Based Blood-Brain Barrier Model for Cytotoxicity Testing and Drug Permeation. Sci. Rep..

[40] Wang X., Xu B., Xiang M., Yang X., Liu Y., Liu X., Shen Y. (2020). Advances on Fluid Shear Stress Regulating Blood-Brain Barrier. Microvasc. Res..

[41] Choublier N., Müller Y., Gomez Baisac L., Laedermann J., de Rham C., Declèves X., Roux A. (2021). Blood–Brain Barrier Dynamic Device with Uniform Shear Stress Distribution for Microscopy and Permeability Measurements. Appl. Sci..

[42] Garcia-Polite F., Martorell J., del Rey-Puech P., Melgar-Lesmes P., O’Brien C.C., Roquer J., Ois A., Principe A., Edelman E.R., Balcells M. (2017). Pulsatility and High Shear Stress Deteriorate Barrier Phenotype in Brain Microvascular Endothelium. J. Cereb. Blood Flow Metab..

[43] de Laere M., Sousa C., Meena M., Buckinx R., Timmermans J.-P., Berneman Z., Cools N. (2017). Increased Transendothelial Transport of CCL3 Is Insufficient to Drive Immune Cell Transmigration through the Blood--Brain Barrier under Inflammatory Conditions In Vitro. Mediators Inflamm..

[44] Meena M., van Delen M., de Laere M., Sterkens A., Costas Romero C., Berneman Z., Cools N. (2021). Transmigration across a Steady-State Blood–Brain Barrie Induces Activation of Circulating Dendritic Cells Partly Mediated by Actin Cytoskeletal Reorganization. Membranes.

[45] Bischel L.L., Coneski P.N., Lundin J.G., Wu P.K., Giller C.B., Wynne J., Ringeisen B.R., Pirlo R.K. (2016). Electrospun Gelatin Biopapers as Substrate for In Vitro Bilayer Models of Blood− Brain Barrier Tissue. J. Biomed. Mater. Res. A.

[46] Palumbo P., Picchini U., Beck B., van Gelder J., Delbar N., DeGaetano A. (2008). A General Approach to the Apparent Permeability Index. J. Pharmacokinet. Pharmacodyn..

[47] Sánchez A.B., Calpena A.C., Mallandrich M., Clares B. (2019). Validation of an Ex Vivo Permeation Method for the Intestinal Permeability of Different BCS Drugs and Its Correlation with Caco-2 In Vitro Experiments. Pharmaceutics.

[48] Ozeki K., Kato M., Sakurai Y., Ishigai M., Kudo T., Ito K. (2015). Evaluation of the Appropriate Time Range for Estimating the Apparent Permeability Coefficient (Papp) in a Transcellular Transport Study. Int. J. Pharm..

[49] Malek A.M., Izumo S. (1996). Mechanism of Endothelial Cell Shape Change and Cytoskeletal Remodeling in Response to Fluid Shear Stress. J. Cell Sci..

[50] Fisher A.B., Chien S., Barakat A.I., Nerem R.M. (2001). Endothelial Cellular Response to Altered Shear Stress. Am. J. Physiol.-Lung Cell. Mol. Physiol..

[51] Ballermann B.J., Dardik A., Eng E., Liu A. (1998). Shear Stress and the Endothelium. Kidney Int..

[52] Tornavaca O., Chia M., Dufton N., Almagro L.O., Conway D.E., Randi A.M., Schwartz M.A., Matter K., Balda M.S. (2015). ZO-1 Controls Endothelial Adherens Junctions, Cell–Cell Tension, Angiogenesis, and Barrier Formation. J. Cell Biol..

[53] Bauer H.-C., Krizbai I.A., Bauer H., Traweger A. (2014). “You Shall Not Pass”—Tight Junctions of the Blood Brain Barrier. Front. Neurosci..

[54] Balda M.S., Matter K. (2009). Tight Junctions and the Regulation of Gene Expression. Biochim. Biophys. Acta (BBA)-Biomembr..

[55] Bauer H., Zweimueller-Mayer J., Steinbacher P., Lametschwandtner A., Bauer H.-C. (2010). The Dual Role of Zonula Occludens (ZO) Proteins. J. Biomed. Biotechnol..

[56] Gericke B., Borsdorf S., Wienböker I., Noack A., Noack S., Löscher W. (2021). Similarities and Differences in the Localization, Trafficking, and Function of P-Glycoprotein in MDR1-EGFP-Transduced Rat versus Human Brain Capillary Endothelial Cell Lines. Fluids Barriers CNS.

[57] Grant G.A., Abbott N.J., Janigro D. (1998). Understanding the Physiology of the Blood-Brain Barrier: In Vitro Models. Physiology.

[58] Kadry H., Noorani B., Cucullo L. (2020). A Blood–Brain Barrier Overview on Structure, Function, Impairment, and Biomarkers of Integrity. Fluids Barriers CNS.

[59] Jamieson J.J., Searson P.C., Gerecht S. (2017). Engineering the Human Blood-Brain Barrier In Vitro. J. Biol. Eng..

[60] Garcia C.M., Darland D.C., Massingham L.J., D’Amore P.A. (2004). Endothelial Cell–Astrocyte Interactions and TGFβ Are Required for Induction of Blood–Neural Barrier Properties. Dev. Brain Res..

[61] Abbott N.J. (2002). Astrocyte–Endothelial Interactions and Blood–Brain Barrier Permeability. J. Anat..

[62] Gaillard P.J., Voorwinden L.H., Nielsen J.L., Ivanov A., Atsumi R., Engman H., Ringbom C., de Boer A.G., Breimer D.D. (2001). Establishment and Functional Characterization of an In Vitro Model of the Blood–Brain Barrier, Comprising a Co-Culture of Brain Capillary Endothelial Cells and Astrocytes. Eur. J. Pharm. Sci..

[63] Kulczar C., Lubin K.E., Lefebvre S., Miller D.W., Knipp G.T. (2017). Development of a Direct Contact Astrocyte-Human Cerebral Microvessel Endothelial Cells Blood–Brain Barrier Coculture Model. J. Pharm. Pharmacol..

[64] Siddharthan V., Kim Y.v., Liu S., Kim K.S. (2007). Human Astrocytes/Astrocyte-Conditioned Medium and Shear Stress Enhance the Barrier Properties of Human Brain Microvascular Endothelial Cells. Brain Res..

[65] Weksler B.B., Subileau E.A., Perriere N., Charneau P., Holloway K., Leveque M., Tricoire-Leignel H., Nicotra A., Bourdoulous S., Turowski P. (2005). Blood-brain Barrier-specific Properties of a Human Adult Brain Endothelial Cell Line. FASEB J..

[66] Raub T.J., Kuentzel S.L., Sawada G.A. (1992). Permeability of Bovine Brain Microvessel Endothelial Cells In Vitro: Barrier Tightening by a Factor Released from Astroglioma Cells. Exp. Cell Res..

[67] Wang C., Baker B.M., Chen C.S., Schwartz M.A. (2013). Endothelial Cell Sensing of Flow Direction. Arter. Thromb. Vasc. Biol..

[68] Islas S., Vega J., Ponce L., González-Mariscal L. (2002). Nuclear Localization of the Tight Junction Protein ZO-2 in Epithelial Cells. Exp. Cell Res..

[69] Traweger A., Fuchs R., Krizbai I.A., Weiger T.M., Bauer H.-C., Bauer H. (2003). The Tight Junction Protein ZO-2 Localizes to the Nucleus and Interacts with the Heterogeneous Nuclear Ribonucleoprotein Scaffold Attachment Factor-B. J. Biol. Chem..

[70] Jaramillo B.E., Ponce A., Moreno J., Betanzos A., Huerta M., Lopez-Bayghen E., Gonzalez-Mariscal L. (2004). Characterization of the Tight Junction Protein ZO-2 Localized at the Nucleus of Epithelial Cells. Exp. Cell Res..

[71] Wu L.-W., Yin F., Peng J., Wang W.-D., Gan N. (2008). The Tight Junction Proteins ZO-1, Occludin and Actin Participate in the Permeability Increasing of Blood-Brain Barrier Induced by Hypoxia-Ischemia. Zhongguo Dang Dai Er Ke Za Zhi.

[72] Hashimoto Y., Campbell M. (2020). Tight Junction Modulation at the Blood-Brain Barrier: Current and Future Perspectives. Biochim. Biophys. Acta (BBA)-Biomembr..

[73] Lochhead J.J., Yang J., Ronaldson P.T., Davis T.P. (2020). Structure, Function, and Regulation of the Blood-Brain Barrier Tight Junction in Central Nervous System Disorders. Front. Physiol..

[74] Liu W., Wang Z., Zhang L., Wei X., Li L. (2012). Tight Junction in Blood-brain Barrier: An Overview of Structure, Regulation, and Regulator Substances. CNS Neurosci. Ther..

[75] Wolburg H., Lippoldt A. (2002). Tight Junctions of the Blood–Brain Barrier: Development, Composition and Regulation. Vascul. Pharmacol..

[76] Huber J.D., Egleton R.D., Davis T.P. (2001). Molecular Physiology and Pathophysiology of Tight Junctions in the Blood–Brain Barrier. Trends Neurosci..

[77] Jóźwik A., Frymus T. (2005). Comparison of the Immunofluorescence Assay with RT-PCR and Nested PCR in the Diagnosis of Canine Distemper. Vet. Res. Commun..

[78] Reis A.D., Fink M.C.D., Machado C.M., Paz J.d.P., Oliveira R.R., Tateno A.F., Machado A.F., Cardoso M.R., Pannuti C.S. (2008). Comparison of Direct Immunofluorescence, Conventional Cell Culture and Polymerase Chain Reaction Techniques for Detecting Respiratory Syncytial Virus in Nasopharyngeal Aspirates from Infants. Rev. Inst. Med. Trop. Sao Paulo.

[79] Vaidya V.M., Malik S.V.S., Kaur S., Kumar S., Barbuddhe S.B. (2008). Comparison of PCR, Immunofluorescence Assay, and Pathogen Isolation for Diagnosis of Q Fever in Humans with Spontaneous Abortions. J. Clin. Microbiol..

[80] Chotiprasitsakul D., Pewloungsawat P., Setthaudom C., Santanirand P., Pornsuriyasak P. (2020). Performance of Real-Time PCR and Immunofluorescence Assay for Diagnosis of Pneumocystis Pneumonia in Real-World Clinical Practice. PLoS ONE.

[81] Urich E., Lazic S.E., Molnos J., Wells I., Freskgård P.-O. (2012). Transcriptional Profiling of Human Brain Endothelial Cells Reveals Key Properties Crucial for Predictive In Vitro Blood-Brain Barrier Models. PLoS ONE.

[82] Lutzky V.P., Carnevale R.P., Alvarez M.J., Maffia P.C., Zittermann S.I., Podhajcer O.L., Issekutz A.C., Chuluyan H.E. (2006). Platelet-endothelial Cell Adhesion Molecule-1 (CD31) Recycles and Induces Cell Growth Inhibition on Human Tumor Cell Lines. J. Cell Biochem..

[83] Clark P.R., Manes T.D., Pober J.S., Kluger M.S. (2007). Increased ICAM-1 Expression Causes Endothelial Cell Leakiness, Cytoskeletal Reorganization and Junctional Alterations. J. Investig. Dermatol..

[84] Cucullo L., Couraud P.-O., Weksler B., Romero I.-A., Hossain M., Rapp E., Janigro D. (2008). Immortalized Human Brain Endothelial Cells and Flow-Based Vascular Modeling: A Marriage of Convenience for Rational Neurovascular Studies. J. Cereb. Blood Flow Metab..

[85] Arisaka T., Mitsumata M., Kawasumi M., Tohjima T., Hirose S., Yoshida Y. (1995). Effects of Shear Stress on Glycosaminoglycan Synthesis in Vascular Endothelial Cells. Ann. N. Y. Acad. Sci..

[86] Santaguida S., Janigro D., Hossain M., Oby E., Rapp E., Cucullo L. (2006). Side by Side Comparison between Dynamic versus Static Models of Blood–Brain Barrier In Vitro: A Permeability Study. Brain Res..

[87] Butt A.M., Jones H.C., Abbott N.J. (1990). Electrical Resistance across the Blood-brain Barrier in Anaesthetized Rats: A Developmental Study. J. Physiol..

[88] Crone C., Olesen S.P. (1982). Electrical Resistance of Brain Microvascular Endothelium. Brain Res..

[89] Xu H., Li Z., Yu Y., Sizdahkhani S., Ho W.S., Yin F., Wang L., Zhu G., Zhang M., Jiang L. (2016). A Dynamic In Vivo-like Organotypic Blood-Brain Barrier Model to Probe Metastatic Brain Tumors. Sci. Rep..

[90] Cucullo L., Marchi N., Hossain M., Janigro D. (2011). A Dynamic In Vitro BBB Model for the Study of Immune Cell Trafficking into the Central Nervous System. J. Cereb. Blood Flow Metab..

[91] Tsuji A., Tamai I. (1997). Blood-Brain Barrier Function of P-Glycoprotein. Adv. Drug Deliv. Rev..

[92] Noack A., Noack S., Buettner M., Naim H.Y., Löscher W. (2016). Intercellular Transfer of P-Glycoprotein in Human Blood-Brain Barrier Endothelial Cells Is Increased by Histone Deacetylase Inhibitors. Sci. Rep..

[93] Roninson I.B. (1992). The Role of the MDR1 (P-Glycoprotein) Gene in Multidrug Resistance In Vitro and In Vivo. Biochem. Pharmacol..

[94] Schinkel A.H., Smit J.J.M., van Tellingen M., Beijnen J.H., Wagenaar E., van Deemter L., Mol C., van der Valk M.A., Robanus-Maandag E.C., te Riele H.P.J. (1994). Disruption of the Mouse Mdr1a P-Glycoprotein Gene Leads to a Deficiency in the Blood-Brain Barrier and to Increased Sensitivity to Drugs. Cell.

[95] Bart J., Willemsen A.T.M., Groen H.J.M., van der Graaf W.T.A., Wegman T.D., Vaalburg W., de Vries E.G.E., Hendrikse N.H. (2003). Quantitative Assessment of P-Glycoprotein Function in the Rat Blood–Brain Barrier by Distribution Volume of [11C] Verapamil Measured with PET. Neuroimage.

[96] Gaillard P.J., van der Sandt I.C.J., Voorwinden L.H., Vu D., Nielsen J.L., de Boer A.G., Breimer D.D. (2000). Astrocytes Increase the Functional Expression of P-Glycoprotein in an In Vitro Model of the Blood-Brain Barrier. Pharm. Res..

[97] Eisenblätter T., Hüwel S., Galla H.-J. (2003). Characterisation of the Brain Multidrug Resistance Protein (BMDP/ABCG2/BCRP) Expressed at the Blood–Brain Barrier. Brain Res..

[98] Austin Doyle L., Ross D.D. (2003). Multidrug Resistance Mediated by the Breast Cancer Resistance Protein BCRP (ABCG2). Oncogene.

[99] Johnsen K.B., Burkhart A., Melander F., Kempen P.J., Vejlebo J.B., Siupka P., Nielsen M.S., Andresen T.L., Moos T. (2017). Targeting Transferrin Receptors at the Blood-Brain Barrier Improves the Uptake of Immunoliposomes and Subsequent Cargo Transport into the Brain Parenchyma. Sci. Rep..

[100] Jefferies W.A., Brandon M.R., Hunt S.v., Williams A.F., Gatter K.C., Mason D.Y. (1984). Transferrin Receptor on Endothelium of Brain Capillaries. Nature.

[101] Santa-Maria A.R., Walter F.R., Figueiredo R., Kincses A., Vigh J.P., Heymans M., Culot M., Winter P., Gosselet F., Dér A. (2021). Flow Induces Barrier and Glycocalyx-Related Genes and Negative Surface Charge in a Lab-on-a-Chip Human Blood-Brain Barrier Model. J. Cereb. Blood Flow Metab..

[102] Zakharova M., do Carmo M.A.P., van der Helm M.W., de Graaf M.N.S., Orlova V., van den Berg A., van der Meer A.D., Broersen K., Segerink L.I. (2020). Multiplexed Blood–Brain Barrier Organ-on-Chip. Lab Chip.

[103] Raimondi I., Izzo L., Tunesi M., Comar M., Albani D., Giordano C. (2020). Organ-on-a-Chip In Vitro Models of the Brain and the Blood-Brain Barrier and Their Value to Study the Microbiota-Gut-Brain Axis in Neurodegeneration. Front. Bioeng. Biotechnol..

[104] Williams-Medina A., Deblock M., Janigro D. (2021). In Vitro Models of the Blood–Brain Barrier: Tools in Translational Medicine. Front. Med. Technol..

[105] Liang Y., Yoon J.-Y. (2021). In Situ Sensors for Blood-Brain Barrier (BBB) on a Chip. Sens. Actuators Rep..

[106] Linville R.M., Searson P.C. (2021). Next-Generation In Vitro Blood–Brain Barrier Models: Benchmarking and Improving Model Accuracy. Fluids Barriers CNS.

[107] van der Helm M.W., van der Meer A.D., Eijkel J.C.T., van den Berg A., Segerink L.I. (2016). Microfluidic Organ-on-Chip Technology for Blood-Brain Barrier Research. Tissue Barriers.

[108] Cabezas R., Ávila M., Gonzalez J., El-Bachá R.S., Báez E., García-Segura L.M., Jurado Coronel J.C., Capani F., Cardona-Gomez G.P., Barreto G.E. (2014). Astrocytic Modulation of Blood Brain Barrier: Perspectives on Parkinson’s Disease. Front. Cell Neurosci..

[109] Török O., Schreiner B., Schaffenrath J., Tsai H.-C., Maheshwari U., Stifter S.A., Welsh C., Amorim A., Sridhar S., Utz S.G. (2021). Pericytes Regulate Vascular Immune Homeostasis in the CNS. Proc. Natl. Acad. Sci. USA.

[110] Zhang S., Wan Z., Kamm R.D. (2021). Vascularized Organoids on a Chip: Strategies for Engineering Organoids with Functional Vasculature. Lab Chip.

[111] di Lullo E., Kriegstein A.R. (2017). The Use of Brain Organoids to Investigate Neural Development and Disease. Nat. Rev. Neurosci..

[112] Shi Y., Inoue H., Wu J.C., Yamanaka S. (2017). Induced Pluripotent Stem Cell Technology: A Decade of Progress. Nat. Rev. Drug Discov..

[113] Workman M.J., Svendsen C.N. (2020). Recent Advances in Human IPSC-Derived Models of the Blood–Brain Barrier. Fluids Barriers CNS.

[114] Delsing L., Herland A., Falk A., Hicks R., Synnergren J., Zetterberg H. (2020). Models of the Blood-Brain Barrier Using IPSC-Derived Cells. Mol. Cell. Neurosci..

[115] Vatine G.D., Barrile R., Workman M.J., Sances S., Barriga B.K., Rahnama M., Barthakur S., Kasendra M., Lucchesi C., Kerns J. (2019). Human IPSC-Derived Blood-Brain Barrier Chips Enable Disease Modeling and Personalized Medicine Applications. Cell Stem Cell.

[116] Linville R.M., DeStefano J.G., Sklar M.B., Xu Z., Farrell A.M., Bogorad M.I., Chu C., Walczak P., Cheng L., Mahairaki V. (2019). Human IPSC-Derived Blood-Brain Barrier Microvessels: Validation of Barrier Function and Endothelial Cell Behavior. Biomaterials.

[117] Nishihara H., Gastfriend B.D., Soldati S., Perriot S., Mathias A., Sano Y., Shimizu F., Gosselet F., Kanda T., Palecek S.P. (2020). Advancing Human Induced Pluripotent Stem Cell-derived Blood-brain Barrier Models for Studying Immune Cell Interactions. FASEB J..

[118] Mossu A., Rosito M., Khire T., Li Chung H., Nishihara H., Gruber I., Luke E., Dehouck L., Sallusto F., Gosselet F. (2019). A Silicon Nanomembrane Platform for the Visualization of Immune Cell Trafficking across the Human Blood–Brain Barrier under Flow. J. Cereb. Blood Flow Metab..

[119] Cecchelli R., Aday S., Sevin E., Almeida C., Culot M., Dehouck L., Coisne C., Engelhardt B., Dehouck M.-P., Ferreira L. (2014). A Stable and Reproducible Human Blood-Brain Barrier Model Derived from Hematopoietic Stem Cells. PLoS ONE.

[120] Qian T., Maguire S.E., Canfield S.G., Bao X., Olson W.R., Shusta E.v., Palecek S.P. (2017). Directed Differentiation of Human Pluripotent Stem Cells to Blood-Brain Barrier Endothelial Cells. Sci. Adv..

[121] Jones H.C., Keep R.F., Butt A.M. (1992). The Development of Ion Regulation at the Blood-Brain Barrier. Prog. Brain Res..

[122] Gendelman H.E., Ding S., Gong N., Liu J., Ramirez S.H., Persidsky Y., Mosley R.L., Wang T., Volsky D.J., Xiong H. (2009). Monocyte Chemotactic Protein-1 Regulates Voltage-Gated K+ Channels and Macrophage Transmigration. J. Neuroimmune Pharmacol..

[123] Breschi G.L., Cametti M., Mastropietro A., Librizzi L., Baselli G., Resnati G., Metrangolo P., de Curtis M. (2013). Different Permeability of Potassium Salts across the Blood-Brain Barrier Follows the Hofmeister Series. PLoS ONE.

[124] Miah M.K., Chowdhury E.A., Bickel U., Mehvar R. (2017). Evaluation of [14C] and [13C] Sucrose as Blood–Brain Barrier Permeability Markers. J. Pharm. Sci..

[125] Paulson J.R., Roder K.E., McAfee G., Allen D.D., van der Schyf C.J., Abbruscato T.J. (2006). Tobacco Smoke Chemicals Attenuate Brain-to-Blood Potassium Transport Mediated by the Na, K, 2Cl-Cotransporter during Hypoxia-Reoxygenation. J. Pharmacol. Exp. Ther..

[126] Behrens M., Hüwel S., Galla H.-J., Humpf H.-U. (2015). Blood-Brain Barrier Effects of the Fusarium Mycotoxins Deoxynivalenol, 3 Acetyldeoxynivalenol, and Moniliformin and Their Transfer to the Brain. PLoS ONE.

[127] Oppenheim H.A., Lucero J., Guyot A.-C., Herbert L.M., McDonald J.D., Mabondzo A., Lund A.K. (2013). Exposure to Vehicle Emissions Results in Altered Blood Brain Barrier Permeability and Expression of Matrix Metalloproteinases and Tight Junction Proteins in Mice. Part. Fibre Toxicol..

[128] Huber J.D., Hau V.S., Borg L., Campos C.R., Egleton R.D., Davis T.P. (2002). Blood-Brain Barrier Tight Junctions Are Altered during a 72-h Exposure to λ-Carrageenan-Induced Inflammatory Pain. Am. J. Physiol.-Heart Circ. Physiol..

[129] Hawkins B.T., Sykes D.B., Miller D.S. (2010). Rapid, Reversible Modulation of Blood–Brain BarrierP-Glycoprotein Transport Activity by Vascular Endothelial Growth Factor. J. Neurosci..

[130] Ronaldson P.T., DeMarco K.M., Sanchez-Covarrubias L., Solinsky C.M., Davis T.P. (2009). Transforming Growth Factor-β Signaling Alters Substrate Permeability and Tight Junction Protein Expression at the Blood-Brain Barrier during Inflammatory Pain. J. Cereb. Blood Flow Metab..

[131] Bickel U., Schumacher O.P., Kang Y.-S., Voigt K. (1996). Poor Permeability of Morphine 3-Glucuronide and Morphine 6-Glucuronide through the Blood-Brain Barrier in the Rat. J. Pharmacol. Exp. Ther..

[132] Ziylan Y.Z., LeFauconnier J.M., Bernard G., Bourre J.M. (1988). Effect of Dexamethasone on Transport of A-Aminoisobutyric Acid and Sucrose Across the Blood-Brain Barrier. J. Neurochem..

[133] Yin D., Wang X., Konda B.M., Ong J.M., Hu J., Sacapano M.R., Ko M.K., Espinoza A.J., Irvin D.K., Shu Y. (2008). Increase in Brain Tumor Permeability in Glioma-Bearing Rats with Nitric Oxide Donors. Clin. Cancer Res..

[134] Jin L., Nation R.L., Li J., Nicolazzo J.A. (2013). Species-Dependent Blood-Brain Barrier Disruption of Lipopolysaccharide: Amelioration by Colistin In Vitro and In Vivo. Antimicrob. Agents Chemother..

